# Cryo-EM structure of the nuclear ring from *Xenopus laevis* nuclear pore complex

**DOI:** 10.1038/s41422-021-00610-w

**Published:** 2022-02-17

**Authors:** Gaoxingyu Huang, Xiechao Zhan, Chao Zeng, Xuechen Zhu, Ke Liang, Yanyu Zhao, Pan Wang, Qifan Wang, Qiang Zhou, Qinghua Tao, Minhao Liu, Jianlin Lei, Chuangye Yan, Yigong Shi

**Affiliations:** 1 Westlake Laboratory of Life Sciences and Biomedicine, 18 Shilongshan Road, Hangzhou, Zhejiang China; 2 Key Laboratory of Structural Biology of Zhejiang Province, School of Life Sciences, Westlake University, 18 Shilongshan Road, Hangzhou, Zhejiang China; 3 Institute of Biology, Westlake Institute for Advanced Study, 18 Shilongshan Road, Hangzhou, Zhejiang China; 4 Beijing Advanced Innovation Center for Structural Biology & Frontier Research Center for Biological Structure, Tsinghua University, Beijing, China; 5 Tsinghua University-Peking University Joint Center for Life Sciences; School of Life Sciences, Tsinghua University, Beijing, China

**Keywords:** Cryoelectron microscopy, Nuclear envelope

## Abstract

Nuclear pore complex (NPC) shuttles cargo across the nuclear envelope. Here we present single-particle cryo-EM structure of the nuclear ring (NR) subunit from *Xenopus laevis* NPC at an average resolution of 5.6 Å. The NR subunit comprises two 10-membered Y complexes, each with the nucleoporin ELYS closely associating with Nup160 and Nup37 of the long arm. Unlike the cytoplasmic ring (CR) or inner ring (IR), the NR subunit contains only one molecule each of Nup205 and Nup93. Nup205 binds both arms of the Y complexes and interacts with the stem of inner Y complex from the neighboring subunit. Nup93 connects the stems of inner and outer Y complexes within the same NR subunit, and places its N-terminal extended helix into the axial groove of Nup205 from the neighboring subunit. Together with other structural information, we have generated a composite atomic model of the central ring scaffold that includes the NR, IR, and CR. The IR is connected to the two outer rings mainly through Nup155. This model facilitates functional understanding of vertebrate NPC.

## Introduction

**Fig. 1:**
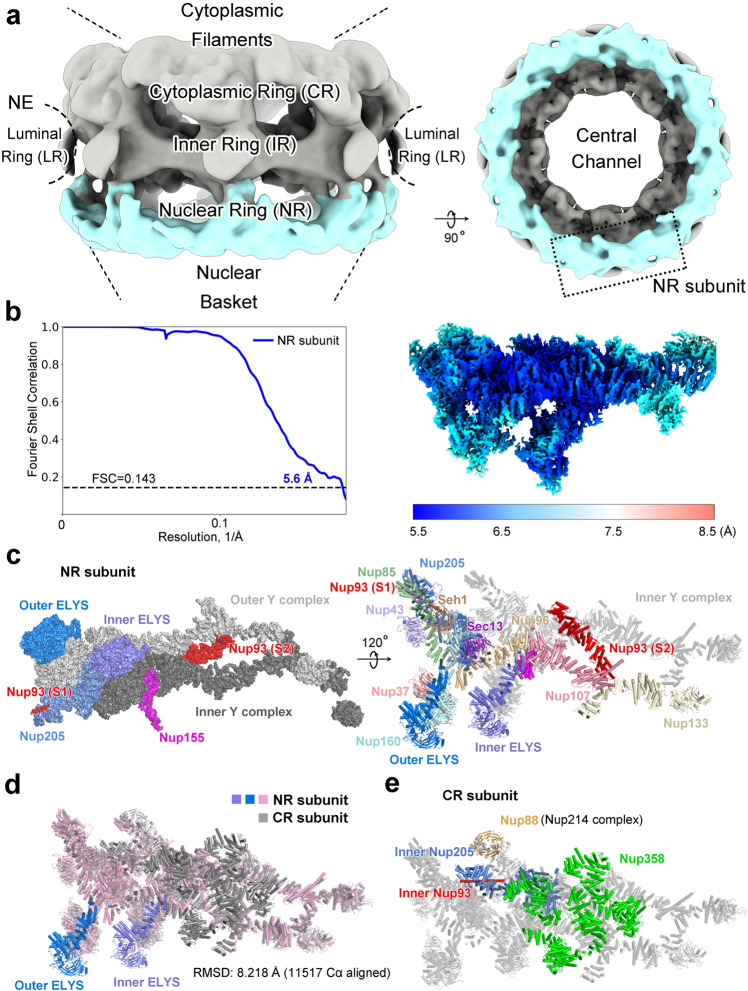
Cryo-EM structure of the NR subunit of the *X. laevis* NPC. **a** A cartoon diagram of the central ring scaffolds of the NPC. The central ring scaffolds consist of the CR, IR, and NR. Cytoplasmic filaments and nuclear basket are connected to the CR and NR, respectively. Two views are shown to highlight the NR (colored cyan). **b** Cryo-EM reconstruction of the NR subunit at an average resolution of 5.6 Å. The Fourier Shell Correlation (FSC) over resolution and distribution of local resolution for the EM reconstruction are shown in the left and right panels, respectively. **c** Structure of the NR subunit from *X. laevis* NPC. Two related views are shown. Individual nucleoporins are shown in surface representation (left panel) and as color-coded cartoon (right panel). The structurally resolved NR subunit contains 22 molecules of 12 distinct nucleoporins, one molecule each for Nup205/Nup93 and two molecules each for the other 10 nucleoporins. The latter 20 nucleoporins constitute two Y complexes. Compared to the CR subunit, each Y complex in the NR subunit associates with one molecule of ELYS. Nup155 comes from the IR subunit. **d** Structure comparison between the NR and CR subunits from *X. laevis* NPC. Two molecules of ELYS are uniquely present in the NR subunit, but not the CR subunit. The two Y complexes of the NR subunit can be superimposed with those of the CR subunit with an RMSD of ~8.2 Å over 11,517 aligned Cα atoms. **e** Structure of the CR subunit from *X. laevis* NPC. The nucleoporins that are absent in the NR subunit are color-coded: five molecules of Nup358 (green), inner Nup205 (marine), an additional molecule of Nup93 (red), and Nup88 (orange) of the Nup214 complex.

Nuclear pore complex (NPC) constitutes the only regular route of cargo transport between the cytoplasm and the nucleus.^1,2^ NPC resides on the nuclear envelope (NE) of all eukaryotic cells. Although the size and composition of the NPC may vary across species,^3–7^ the overall organization is conserved among all eukaryotes.^2,8–10^ The vertebrate NPC, with a combined molecular mass of over 100 MDa,^11–13^ consists of three central ring scaffolds that collectively define the central pore: cytoplasmic ring (CR), inner ring (IR), and nuclear ring (NR)^4,9,14,15^ (Fig. 1a). CR and NR share a similar overall organization and are characterized by a conserved set of nucleoporins.^15^ IR in the center of the NPC is sandwiched by two outer rings (CR and NR) and displays a two-fold symmetry.^16,17^

Structural investigation of the NPC has yielded rich information on various ring scaffolds^3,4,6,7,10,18^ and their constituent nucleoporins.^4,9,19,20^ At present, the EM reconstruction through cryo-electron tomography (cryo-ET) has been generated at low-to-medium resolution for human NPC^21^ and for *Xenopus laevis* (*X. laevis*) NPC.^22^ Composite coordinates for human NPC were generated by docking known X-ray structures into the EM map.^16,17,21^ Relying on cryo-EM single particle analysis (SPA), we previously reconstructed the CR subunit from *X. laevis* NPC with a local resolution of ~5–8 Å.^23^ We have recently improved the average resolution of the *X. laevis* NPC to 4.1 Å for the CR subunit^24^ and 4.2 Å for the IR subunit^25^ through improvement in sample preparation and cryo-EM analysis.

In this study, we report the cryo-EM reconstruction of the NR subunit of the *X. laevis* NPC at an average resolution of 5.6 Å, which reaches the resolution limit at the bin-2 level data processing. Features of the EM maps permit docking of known and predicted structures of *X. laevis* nucleoporins^24–26^ and identification of secondary structural elements in most nucleoporins of the NR subunit. Based on the EM maps, we have generated atomic coordinates for 22 nucleoporins of the *X. laevis* NR subunit, which include 18,941 amino acids. Together with reconstructions of the CR and IR subunits, we report EM map-based atomic coordinates of the central ring scaffolds of *X. laevis* NPC. This structure information serves as a framework for mechanistic understanding of NPC assembly and function in vertebrates.

## Results

### Cryo-EM analysis of the *X. laevis* NR subunit

We reconstructed the NR subunit of the *X. laevis* NPC using the same EM dataset as that for reconstruction of the CR subunit.^24^ Relying on 660,302 NPC particles, we first reconstructed the NR subunit at an initial resolution of 22 Å with C8 symmetry (Supplementary information, Fig. S1a). The overall structure is similar to that of the NR from human NPC^21^ (Supplementary information, Fig. S1b). This reconstruction allowed extraction of 4.4 million individual NR subunits (Supplementary information, Fig. S2). Using data at the bin-2 level, we generated a reconstruction of the NR subunit, which after auto-refinement displays an average resolution of 5.6 Å based on 813,020 particles (Fig. 1b; Supplementary information, Figs. S2, S3 and Tables S1, S2).

Our cryo-EM reconstruction of the *X. laevis* NR subunit reveals clear features of secondary structural elements (Supplementary information, Figs. S4–S7). The improved EM density maps allowed assignment of most components in the NR subunit and placement of secondary structural elements. We first docked the atomic coordinates of inner and outer Y complexes from the CR subunit^24^ into the EM map (Supplementary information, Figs. S4–S6). Next, we placed Nup93 and Nup205 into the map. These practices leave a well-defined chunk of EM density along the long arm of inner Y complex, which nicely accommodates the AlphaFold-predicted structure of the *X. laevis* nucleoporin ELYS^26^ (Supplementary information, Fig. S7a). A similarly-shaped EM density of lower resolution along the long arm of outer Y complex was assigned to a second molecule of ELYS (Supplementary information, Fig. S7b). Altogether, we identified 22 molecules of 12 distinct nucleoporins in each NR subunit (Fig. 1c). In addition, we also located a portion of Nup155 from the IR subunit and tentatively assigned one molecule each of Nup98 and TPR.

### Overall structure of the NR subunit

Each NR subunit mainly comprises two 10-membered Y complexes. Each Y complex comprises a short arm (Nup85, Nup43, and Seh1), a long arm (Nup160, Nup37, and ELYS), and a stem (Sec13, Nup96, Nup107, and Nup133). Compared to the CR, each Y complex in the NR contains an additional nucleoporin ELYS, which closely associates with Nup160 and Nup37 in the long arm. These two Y complexes are connected and stabilized by two key nucleoporins Nup93 and Nup205. Altogether, the final atomic model of the *X. laevis* NR subunit contains 18,894 amino acids.

With the exception of including ELYS in the long arm, the overall organization of the two Y complexes in the NR subunit is similar to that in the CR subunit, with most variations occurring at the interfaces with adjacent subunits or NR/CR-specific nucleoporins. The two Y complexes of the NR subunit as a whole can be superimposed to those of the CR subunit with a root-mean-squared deviation (RMSD) of 8.22 Å over 11,517 aligned Cα atoms (Fig. 1d). Notably, however, compared to the NR subunit, the CR subunit contains an extra molecule of Nup93, an extra molecule of Nup205 (inner Nup205), and five molecules of Nup358 (Fig. 1e).

### ELYS associates with the long arm of the Y complex

**Fig. 2:**
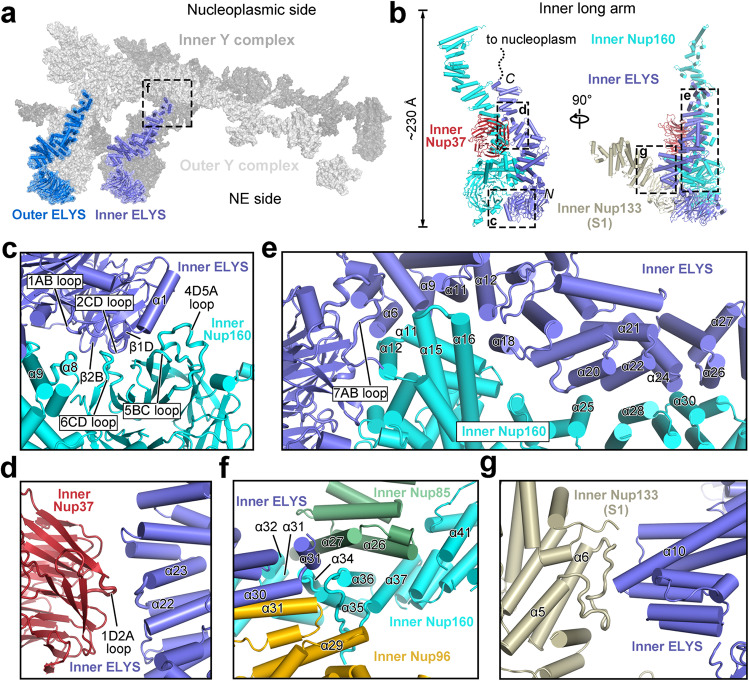
Association of ELYS with the long arm of the Y complex in *X. laevis* NR. **a** The N-terminal domain of ELYS associates with the long arm of the Y complex in the NR subunit. The outer ELYS (marine) and inner ELYS (purple) associate with the outer and inner Y complexes, respectively. **b** Structure of the long arm of the inner Y complex. The long arm, spanning a length of ~23 nm, consists of Nup160, Nup37, and ELYS. Inner ELYS from one NR subunit also associates with inner Nup133 from an adjacent subunit. **c** A close-up view on the interface between the two N-terminal β-propellers of inner ELYS and inner Nup160. **d** A close-up view on the interface between the α-solenoid of ELYS and Nup37. **e** A close-up view on the extended interface between the α-helical domain of Nup160 and ELYS. In short, eight helices and three loops from inner ELYS engage in interactions with structural elements from inner Nup160, including seven helices from the inner Nup160 α-helical domain. **f** A close-up view on the interactions of the C-terminal α-helices of inner ELYS with the C-terminal α-helices of inner Nup85, inner Nup96, and inner Nup160. **g** A close-up view on the interface between the α-helical domain of inner ELYS and the α-helical domain of inner Nup133 from the adjacent subunit. Helix α10 of inner ELYS closely interacts with the loop between α5 and α6 of inner Nup133. All loops involved in the interaction between nucleoporins are highlighted in thicker form.

The primary components of the NR subunit are inner and outer Y complexes: the former closer to the NPC central pore than the latter. Unlike the CR subunit,^24^ the long arm of each Y complex in the NR subunit closely associates with one ELYS molecule (Fig. 2a). ELYS is reported to recruit the Y complex to the chromatin, initializing the postmitotic reassembly of NPC in metazoans.^27,28^ The β-propeller and α-helical domain in the N-terminal half of ELYS bind to the long arm of Y complex in the NR,^29^ whereas the AT-hook domain in the intrinsically disordered C-terminal half exhibits high affinity to the chromatin.^30^

In our structure, the N-terminal β-propeller of inner ELYS stacks against the N-terminal β-propeller of inner Nup160; the α-helical domain of inner ELYS aligns with the α-helical domain of inner Nup160, orienting the AT-hook domain into the nucleoplasm. Similarly, outer ELYS associates with outer Nup160 in an almost identical manner. Through close interactions with Nup160 and Nup37, ELYS becomes an integral component of the long arm of the Y complex in the NR subunit (Fig. 2b).

Specifically, at the interface between the two N-terminal β-propellers of ELYS and Nup160, two β-strands (β1D and β2B) and the 2CD loop (the loop between strands C and D in the blade 2) of inner ELYS associate with the 6CD loop of inner Nup160 (Fig. 2c). The 1AB loop of inner ELYS interacts with the loop between α8 and α9 of inner Nup160. In addition, helix α1 of inner ELYS binds the 4D5A and 5BC loops of inner Nup160. At the interface between the ELYS α-helical domain and the Nup37 β-propeller, the loop between α22 and α23 of inner ELYS associates with the 1D2A loop on the top face of the β-propeller (Fig. 2d). At one end of the extended interface between the α-helical domains of Nup160 and ELYS, the 7AB loop of inner ELYS binds helix α12 of inner Nup160 (Fig. 2e). Helices α6, α9, α11, and α12/α18 of inner ELYS associate with α11/α12, α11, the α15–α16 loop, α16 of inner Nup160, respectively. At the other end of the interface, the α20–α21 loop and α22/α24 of inner ELYS bind α25 and α28 of inner Nup160, respectively. Helix α24 and the α26–α27 loop of inner ELYS contact helix α30 of inner Nup160 (Fig. 2e).

The C-terminal α-helices of inner ELYS α-helical domain are positioned in the center of the vertex of the Y complex and directly interact with the C-terminal helices of inner Nup85, inner Nup96, and inner Nup160 (Fig. 2f). Specifically, helix α31 of inner ELYS contacts helix α27 of inner Nup85. Helix α30 of inner ELYS interacts with helix α31 of inner Nup96. In addition, the loop between helices α30 and α31 of inner ELYS is in close proximity to the loop between α34 and α35 of inner Nup160. Notably, inner ELYS from one NR subunit also bridges interaction with an adjacent NR subunit (Fig. 2g). Helix α10 of inner ELYS closely interacts with the loop between helices α5 and α6 of inner Nup133 from a neighboring NR subunit.

### Nup205 connects the inner and outer Y complexes

**Fig. 3:**
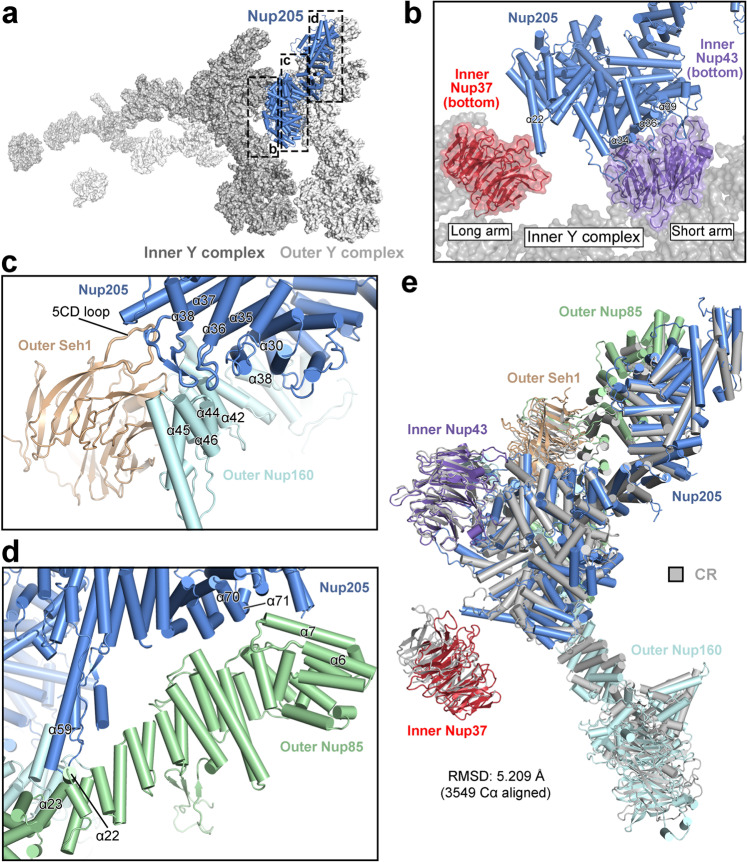
Nup205 connects the inner and outer Y complexes in the NR subunit. **a** Nup205 connects the inner and outer Y complexes. Nup205 interacts with the short arm and the vertex of outer Y complex; it also associates with both arms of inner Y complex. **b** A close-up view on the association between Nup205 and inner Y complex. The association involves inner Nup37 from the long arm and inner Nup43 from the short arm. **c** A close-up view on the interface between the N-terminal domain of Nup205 and the vertex region of outer Y complex. **d** A close-up view on the interface between the C-terminal domain of Nup205 and outer Nup85. **e** Structure alignment of Nup205 and its binding partners from the NR subunit with those from the CR subunit.

Unlike the CR subunit,^24^ the NR subunit contains only one molecule of Nup205, which corresponds to outer Nup205 in the CR subunit. Similar to that in the CR subunit,^24^ Nup205 in the NR subunit interacts with the short arm and the vertex of outer Y complex; it also associates with both arms of inner Y complex (Fig. 3a). Therefore, Nup205 connects the inner and outer Y complexes.

At the interface with inner Y complex, Nup205 associates with two β-propellers, one from inner Nup37 of the long arm and the other from inner Nup43 of the short arm (Fig. 3b). Specifically, helix α22 of Nup205 interacts with the bottom face of inner Nup37. Helices α34, α36 and α39 of Nup205 contact the bottom face of inner Nup43.

At the interface with the vertex region of outer Y complex, helix α30 of Nup205 interacts with helix α38 of outer Nup160 (Fig. 3c). The loop between α35 and α36 of Nup205 is in close proximity to helix α42 and the loop between α44 and α45 of outer Nup160. The loop between α37 and α38 of Nup205 directly contacts both helix α46 of outer Nup160 and the 5CD loop of outer Seh1. At the interface with the short arm of outer Y complex, Nup205 contacts both ends of outer Nup85 (Fig. 3d). Specifically, the Tower helix α59 of Nup205 associates with helices α22/α23 of outer Nup85. Helices α70 and α71 of Nup205 interact with the loop between α6 and α7 of outer Nup85.

The interfaces involving Nup205 in the NR subunit closely resemble those involving outer Nup205 in the CR subunit.^24^ In fact, Nup205 and its binding partners in the NR can be superimposed to their counterparts in the CR with an RMSD of ~5.2 Å over 3549 aligned Cα atoms (Fig. 3e).

### Nup93 bridges the stems of the Y complexes

**Fig. 4:**
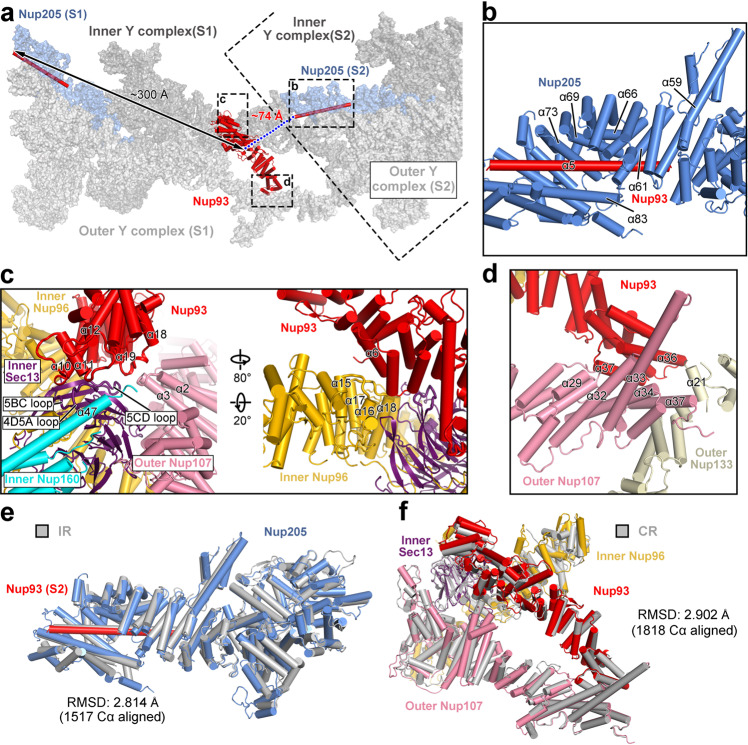
Nup93 bridges the stems of inner and outer Y complexes in the NR subunit. **a** Nup93 bridges the stems of inner and outer Y complexes. The NR subunit contains one molecule each of Nup93 and Nup205. The extended N-terminal helix from Nup93 in subunit 1 (S1) may interact with Nup205 in the neighboring subunit (S2). **b** A close-up view on the interface between Nup93 and Nup205. Helix α5 of Nup93 traverses through the axial groove of the Nup205 α-solenoid. **c** Two close-up views on the interface between Nup93 and the vertex of inner Y complex. **d** A close-up view on the interface between the CTD of Nup93 and the stem of outer Y complex. **e** Structure alignment of Nup205 between the NR and IR subunits. Helix α5 of Nup93 remains bound to Nup205 in both cases. These two structures can be superimposed with an RMSD of ~2.8 Å over 1517 aligned Cα atoms. **f** Structure alignment of Nup93 and its binding partners from the NR subunit with those from the CR subunit. This alignment produces an RMSD of ~2.9 Å over 1818 aligned Cα atoms.

Unlike that in the CR subunit,^24^ the NR subunit only contains one molecule of Nup93, which interacts with both stems of the inner and outer Y complexes (Fig. 4a). The α-solenoid (residues 180–820) of Nup93 is connected to an extended N-terminal helix α5 (residues 101–152) through a flexible sequence of 28 residues. Within the same NR subunit, the distance between residue 180 of the α-solenoid and residue 152 of helix α5 is ~300 Å, which exceeds the maximal distance spanned by residues 152–180. This analysis strongly suggests that the α-solenoid of Nup93 in one NR subunit may place its N-terminal helix α5 into Nup205 in an adjacent NR subunit (Fig. 4a). Consistent with this conclusion, the distance of 74 Å in between can be covered by 28 residues. Similar to that in the IR or CR subunit,^24,25^ helix α5 of Nup93 is placed into the axial groove of Nup205 α-solenoid and interacts with helices α59, α61, α66, α69, α73 and α83 of Nup205 (Fig. 4b).

The two ends of Nup93 α-solenoid mainly associate with the stems of the two Y complexes. At its N-terminal end, helix α10 and the loop between α10 and α11 of Nup93 contact both 4D5A and 5BC loops of inner Sec13 (Fig. 4c, left panel). The loop between α11 and α12 of Nup93 interacts with helix α47 of inner Nup160. The loop between α18 and α19 of Nup93 may simultaneously contact the loop between α2 and α3 of outer Nup107 and the 5CD loop of inner Sec13. Helix α6 of Nup93 binds the loops between α15 and α16 and between α17 and α18 of inner Nup96 (Fig. 4c, right panel). At its C-terminal end, helix α36 of Nup93 contacts helix α32 of outer Nup107 and helix α21 of outer Nup133 (Fig. 4d). Helix α37 of Nup93 binds helices α29, α32, α34 and α37 of outer Nup107.

A recurring theme in the structures of the CR, IR, and NR is association of the Nup93 helix α5 with the axial groove in the CTD of Nup205 α-solenoid. Structure of the α5-bound Nup205 from the NR subunit can be superimposed to that from the IR subunit with an RMSD of ~2.8 Å over 1517 aligned Cα atoms (Fig. 4e). The α-solenoid of Nup93 plays a similar structural role in the CR and NR subunits due to similar arrangement of their Y complexes. Structure of Nup93 and its binding partners from the NR subunit can be superimposed to those from the CR subunit with an RMSD of ~2.9 Å over 1818 aligned Cα atoms (Fig. 4f). In contrast, compared to that in the CR or NR, the α-solenoid of Nup93 interacts with a different set of proteins in the IR subunit.^25^

### Formation of the NR scaffold

**Fig. 5:**
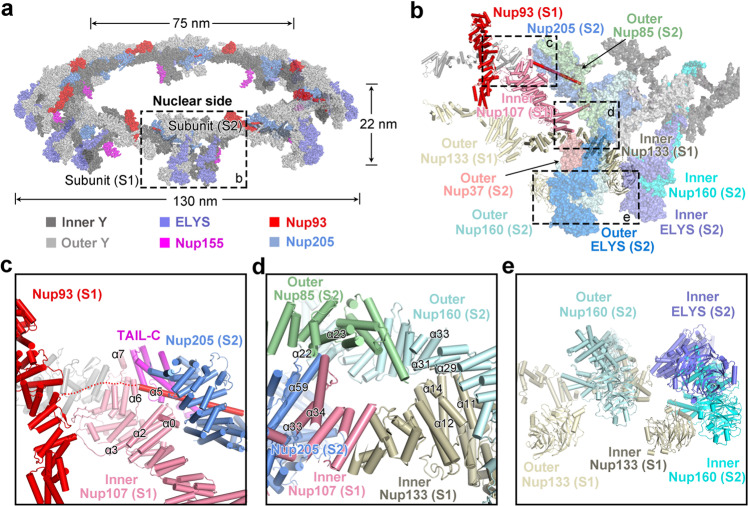
Formation of the NR scaffold. **a** A composite model of the *X. laevis* NR. The *X. laevis* NR has inner and outer diameters of ~75 nm and ~130 nm, respectively. Two neighboring subunits, subunit 1 (S1) and subunit 2 (S2), are labeled by text with S2 highlighted in a dashed box. **b** A close-up view of the boxed region in **a**, showing the clustered interfaces between S1 and S2. The nucleoporins from S1 and S2 are shown as color-coded cartoon and surface representation, respectively. **c** A close-up view on the inter-subunit interface mediated by Nup205 from one subunit (S2) and Nup93 and inner Nup107 from the adjacent subunit (S1). **d** A close-up view on the inter-subunit interface mediated by three nucleoporins (Nup205, outer Nup85 and outer Nup160) from S2 and inner Nup107 and inner Nup133 from S1. **e** A close-up view on the inter-subunit interface mediated by the long arms of the Y complexes from S2 and the stem tips of the Y complexes from S1.

The 5.6-Å reconstruction of the NR subunit was individually aligned to each of the eight subunits in the 22-Å reconstruction of the NR. The atomic coordinates of the NR subunit were individually placed into each of the eight aligned reconstructions, resulting in a composite model of the NR (Fig. 5a). Similar to the CR, eight inner Y complexes and eight outer Y complexes assemble into a proximal ring and a distal ring, respectively.^17^ Unlike that in the CR, each Y complex in the NR contains an extra nucleoporin ELYS, which becomes a component of the long arm. For Nup93 and Nup205, each interacts with both Y complexes within the same NR subunit and mediates interfaces between two neighboring NR subunits. Eight molecules of Nup155 from the IR connect to the NR (Fig. 5a).

The inter-subunit interface involves a number of nucleoporins from both subunits (Fig. 5b). At one end of the interface close to the central pore, the N-terminal helix α5 of Nup93 from one subunit (S1) is placed in the axial groove of Nup205 α-solenoid from an adjacent subunit (S2) (Fig. 5c). Helix α5 of Nup93 also interacts with helix α2 and its ensuing loop of inner Nup107 from S1. In addition, helices α0/α2/α3 of inner Nup107 contact the C-terminal α-solenoid of Nup205 from S2. Helices α6 and α7 of inner Nup107 interact with TAIL-C of Nup205 from S2.

In the center of the inter-subunit interface (Fig. 5d), helices α33 and α34 of inner Nup107 interact with Tower helix α59 of Nup205 from S2 and helices α22/α23 of outer Nup85. Helices α11/α12/α14 of inner Nup133 contact helices α29/α31/α33 of outer Nup160 from S2. At the other end of the inter-subunit interface (Fig. 5e), the long arms of the Y complexes associate with the stem tips of the Y complexes from S1. Specifically, the β-propeller domains of inner and outer Nup133 from S1 interact with the β-propeller domains of inner and outer Nup160 from S2, respectively. Notably, helix α10 of inner ELYS associates with the flexible loop between helices α5 and α6 of inner Nup133 from S1 (Figs. 2g, 5e).

### Linkage of the IR to outer rings through Nup155

**Fig. 6:**
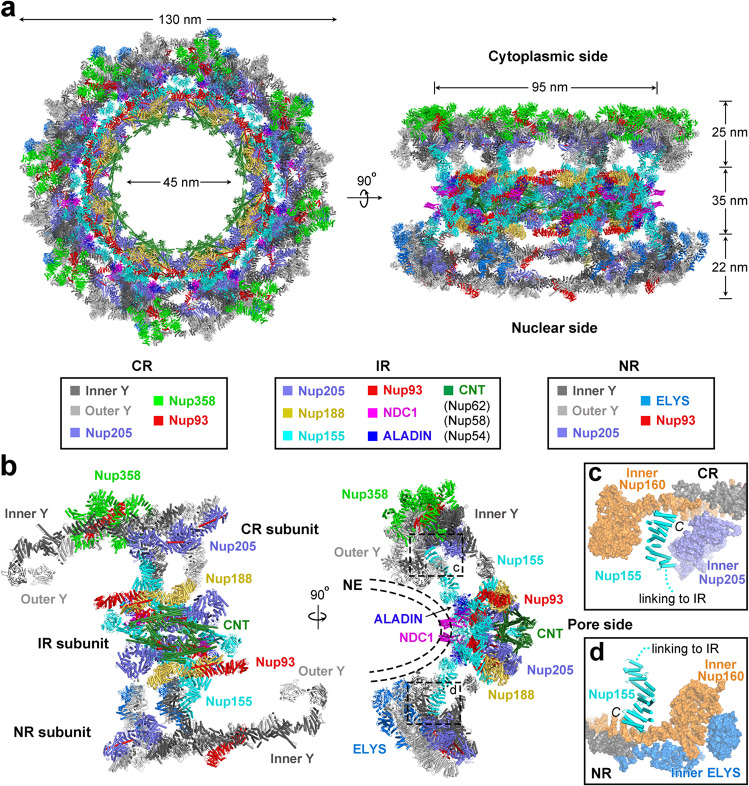
Structure of the central ring scaffold of *X. laevis* NPC. **a** Overall structure of the central ring scaffold (CR/IR/NR) of *X. laevis* NPC. Combining the atomic models of the CR^24^ and IR,^25^ we generated a composite model of the central ring scaffold of *X. laevis* NPC. Shown here are two perpendicular views. All nucleoporins are color-coded and tabulated below the images. The *X. laevis* NPC has outer and inner diameters of ~130 nm and ~45 nm, respectively. **b** Overall structure of one subunit of the central ring scaffold. Two perpendicular views are shown, with nucleoporins color-coded. The CR and NR subunits are placed symmetrically on the cytoplasmic and nuclear sides, respectively, of the symmetric IR subunit. The subunit is anchored on the NE mainly through NDC1 and the β-propeller domains from Nup155, Nup160, Nup133 and ELYS. The CR and NR are linked to the IR mainly through two molecules of Nup155. **c** A close-up view on the interface between the linker Nup155 and CR components. The C-terminal helices of linker Nup155 are sandwiched by inner Nup160 and inner Nup205 from the CR subunit. **d** A close-up view on the interface between linker Nup155 and NR components. The C-terminal helices of linker Nup155 contact the middle portion of the α-helical domain of inner Nup160 from the NR.

We have recently determined the cryo-EM structures of the CR subunit and the IR subunit, both from *X. laevis* NPC, at average resolutions of 4.1 Å^24^ and 4.2 Å,^25^ respectively. In this study, we report the reconstruction of the NR subunit at an average resolution of 5.6 Å (Fig. 1; Supplementary information, Table S1). By aligning the atomic coordinates of the individual subunits back into the NPC particle, we have generated a composite atomic model for the central ring scaffold (CR, IR, and NR) of the *X. laevis* NPC (Fig. 6a). This composite model is based on experimental EM maps that allow accurate identification of secondary structural elements in most nucleoporins. This model contains 632 molecules of 20 distinct nucleoporins, amounting to ~50 MDa in molecular weight.

The central ring scaffold of *X. laevis* NPC has an outer diameter of ~130 nm (Fig. 6a, left panel). In contrast, the outer diameter of the IR is ~95 nm. The considerably smaller outer diameter of the IR compared to the outer rings gives rise to a deep circular groove on the NPC central ring scaffold (Fig. 6a, right panel). This groove allows placement of the NPC onto the convex nuclear pore on the NE (Fig. 6b). Indeed, the 35-nm thickness of the IR agrees well with that of the NE.^31^ CR and NR are slightly thinner, with thicknesses of ~25 nm and ~22 nm, respectively. The central ring scaffold is anchored on the NE mainly through NDC1, and the β-propeller domains of Nup155, Nup160, Nup133 and ELYS (Fig. 6b). The CR and NR are connected to IR mainly through the linker nucleoporin Nup155. Specifically, the C-terminal helices of Nup155 are sandwiched by inner Nup160 and inner Nup205 from the CR subunit on the cytoplasmic side (Fig. 6c). These Nup155 helices contact the middle portion of the α-helical domain of inner Nup160 from the NR subunit (Fig. 6d). Notably, inner Nup205 is absent in the NR subunit.

## Discussion

Detailed structural information is a pre-requisite for mechanistic understanding of NPC function and regulation. Due to the huge size and inherent flexibility of NPC, cryo-ET through sub-tomogram averaging (STA) had been the dominant approach to study the NPC structure. Prior to our studies, the cryo-ET reconstruction for the central ring scaffold of human NPC was generated at a local resolution of up to 15 Å,^21^ the best achieved for vertebrate NPC until 2020. These cryo-ET maps, with domain-level accuracy, allowed rigid-body docking of X-ray structures of various nucleoporins, generating a composite model for human NPC.^16,17,21^ This composite model has greatly advanced our understanding on NPC structure and function. We also took the cryo-ET approach on the *X. laevis* NPC and reconstructed the central ring scaffold at nominally improved resolutions but with some anisotropy.^22^ Importantly, however, our cryo-ET reconstruction reveals detailed features of the luminal ring (LR), which were confirmed by subsequent cryo-EM reconstruction of the LR subunit at 10.7 Å.^22^

We took the cryo-EM SPA approach in structural investigation of the NPC. In 2020, we reported the cryo-EM structure of the CR subunit of *X. laevis* NPC at local resolutions of 5.5–7.9 Å.^23^ Although α-helices appear as featureless tubes in this EM map,^23^ this is the first time secondary structural elements of any nucleoporin are clearly seen in the context of NPC. These EM maps allowed accurate placement of most nucleoporins and resolved some of the ambiguities in the CR.^23^

Continuing our cryo-EM effort, we have most recently determined the reconstructions of the CR and IR subunits at average resolutions of 4.1 Å and 4.2 Å, respectively.^24,25^ In particular, the local resolution in the EM map of the CR subunit reaches 3.8 Å, allowing residue-level assignment of the sequences in select nucleoporins. In this study, we report the reconstruction of the NR subunit at an average resolution of 5.6 Å. The EM maps for the CR, IR, and NR of the *X. laevis* NPC allow us to generate a composite model of the central ring scaffold, which contains ~445,000 amino acids in 632 molecules of nucleoporins (Fig. 6a).

In this model, most secondary structural elements of the nucleoporins are placed into EM density (Supplementary information, Fig. S11). The combined molecular mass of ~50 MDa for this composite model accounts for ~80% of the NPC central ring scaffold. This model serves as a framework for mechanistic understanding of NPC function. Based on this model, we have mapped the domains that are known to anchor the NE onto the central ring scaffold (Supplementary information, Fig. S12). This model may also allow accurate mapping of phosphorylation sites and disease mutations onto the NPC scaffold.

Compared to that of the CR, the Y complex of the NR contains an additional nucleoporin ELYS.^27^ During mitosis of metazoan cells, the NE collapses and the NPC breaks down, resulting in the release of many soluble nucleoporins and subcomplexes into the cytoplasm such as the Y complex.^32^ In anaphase, reassembly of the NPC is initialized by ELYS. With its N-terminal half anchored on the Y complex (Fig. 2), ELYS uses its AT-hook domain at its C-terminal half to associate with the chromatin,^28,30^ leading to formation of the pre-pores and then the intact NPC.^32^ The proximity to NE and exterior placement of ELYS in the NR subunit are fully consistent with its function in NPC reassembly (Fig. 6b).

Nup93 and Nup205 play a key role in all three rings CR/IR/NR of the central ring scaffold. Another nucleoporin Nup188 shares sequence and structural homology with Nup205.^33,34^ In each subunit of the central ring scaffold, there are seven molecules of Nup93, five molecules of Nup205, and two molecules of Nup188. Based on our EM maps, these 14 nucleoporins constitute seven pairs of Nup93–Nup205/Nup188, each with an extended N-terminal helix α5 of Nup93 bound in the axial groove of the α-solenoid of Nup205 or Nup188. One, two, and two pairs of Nup93–Nup205 are present in the NR, IR and CR subunits, respectively. In addition, two pairs of Nup93–Nup188 are present in the IR subunit.

Given the large size of the structured region of Nup205 or Nup188, it is unlikely to place an additional Nup205 or Nup188 molecule into the unassigned EM density. Compared to Nup205 or Nup188, Nup93 is much smaller in size and exhibits considerable conformational flexibility. Although unlikely, we cannot rule out additional molecules of Nup93 in the central ring scaffold of the NPC. In the IR subunit, Nup93 uses its N-terminal sequences to interact with channel nucleoporin heterotrimer (CNT),^35^ Nup188, and Nup205.^25^ In the CR or NR subunit, Nup93 uses two ends of its α-solenoid to bridge inner and outer Y complexes, and places its N-terminal extended helix α5 into the axial groove of Nup205 α-solenoid.

Although the overall resolution for the reconstruction of the NR subunit is 5.6 Å, the local resolution in the peripheral regions drops off rapidly (Fig. 1b). Consequently, two members of the NR subunit — Nup98 and TPR — remain to be conclusively identified. Our current EM maps suggest potential locations for these two nucleoporins (Supplementary information, Fig. S13a). A lobe of EM density below the short arm of inner Y complex likely comes from Nup98 and the target protein is designated as Nup98/X. Nup98 was proposed to be a key linker in formation of the NPC scaffold.^4,9,12^ The C-terminal region of Nup98 autoproteolytic domain (APD) may contact inner Nup85 (Supplementary information, Fig. S13b). This organization contrasts with that in the CR subunit, where the APD of Nup98 interacts with the β-propeller of Nup88 (Supplementary information, Fig. S13c), which in turn contacts the helical ridges of inner Nup85. In addition, the APD of Nup98 may contact the extended helix α5 of inner Nup93 in the CR subunit, which is clearly resolved in our improved local EM map for the Nup214 region of the CR subunit.^24^

Another lobe of EM density, likely coming from TPR (therefore designated as TPR/X), is positioned in close proximity to the long arm of the inner Y complex and the stem of the outer Y complex. Specifically, this protein interacts with outer Nup107, outer Nup96, inner Nup160 and the β-propellers of inner Sec13 and inner Seh1 (Supplementary information, Fig. S13d). A third lobe of unknown EM density is located between the two short arms, occupying the same location as the Nup214 complex of the CR subunit.^23^ These ambiguities in nucleoporin assignment will likely be resolved by additional resolution improvement of the NR subunit.

After completion of this manuscript, we noted online posting of a few related structural studies on the NPC from human, *X. laevis*, and yeast.^36–42^ Advent of the detailed structural information will guide future studies on the function and regulation of vertebrate NPC. In contrast to the central ring scaffold of CR/IR/NR, the LR remains enigmatic and lacks any atomic model. Structure determination of the LR at a higher resolution becomes a pressing task. In the meantime, understanding of the vertebrate central ring scaffold may benefit from structural elucidation of the *X. laevis* NPC at atomic resolutions.

## Materials and methods

### Cryo-EM sample preparation and EM data acquisition

The same cryo-EM dataset used for reconstruction of the CR^24^ and IR^25^ from *X. laevis* oocytes was used for reconstruction of the NR in this study. In short, the NE from *X. laevis* oocytes was prepared as previously described.^22,23^ The gold EM grids (R1.2/1.3, R2/1, and R2/2; Quantifoil, Jena, Germany) were blotted for 8 s with a blot force of 15 and vitrified by plunge-freezing into liquid ethane using Vitrobot Mark IV (Thermo Fisher Scientific) at 8 °C under 100% humidity.

Details of data acquisition are as described, with the grids tilting at angles of 0, 30, 45, and 55 degrees.^24^ A dataset of 46,143 micrographs were recorded on a Titan Krios electron microscope (FEI) operating at 300 kV with a nominal magnification of 64,000× and equipped with a Gatan GIF Quantum energy filter (slit width 20 eV) (Supplementary information, Table S1). A K3 detector (Gatan Company) operating at the super-resolution mode was used for data acquisition, with a calibrated pixel size of 0.6935 Å for the movie files (Supplementary information, Table S1). The movie images were binned twice during motion correction, resulting in a final pixel size of 1.387 Å for the motion corrected images. All frames in each stack were aligned and summed using MotionCor2.^43^ During the alignment process, the raw frames were divided into 13 × 11 patches to perform local alignment and polynomial model estimation.^44^ Dose weighting was performed using MotionCor2.^43^ The average defocus values were set between –1.5 μm and –3.0 μm and per micrograph CTF parameters were estimated using Gctf.^45^

### An initial model of the NR from *X. laevis* NPC

33,747 micrographs were manually selected from the original dataset of 46,143 micrographs for further processing. A total of 800,825 particles were manually selected from these micrographs (Supplementary information, Fig. S1a). Initial per-particle local defocus estimation was carried out as previously described^23^ prior to all other data processing procedures.

The central portion of an NPC comprises four ring scaffolds: CR, IR, NR, and LR. Due to the inherent flexibility among the four rings, it is practically impossible to refine the entire NPC as a single particle to high resolution. We therefore carried out initial pose estimation of the NPC particles on one of the relatively stable ring scaffolds: the CR. The NPC particles were first aligned to the CR side as described.^24^ This procedure allowed selection of 660,302 NPC particles that contributed to the final reconstruction. Following refinement of the CR structure with these 660,302 NPC particles, we further refined the EM density that belongs to other rings using a confined angular and shift search range. We first continued the 3D refinement procedure from the last iteration with a layered mask focusing on the IR and LR layer (the layer immediately adjacent to the CR layer). Only pixels within a specific layer, with *z*_start < *z* < *z*_end, have pixel values of 1; all other pixels that have *z* coordinates below or above this layer have zero values. Transition between these two regions follows a raised cosine scheme. The continue refinement yielded a reconstruction of the IR at 22 Å resolution based on 660,302 NPC particles. The same local refinement strategy was applied again to help refine the EM density that belongs to the NR. Continuing from 3D auto-refinement of the IR, a layered mask focusing on the NR layer was further applied for 3D refinement. A final reconstruction of the NR was finally obtained out of 660,302 NPC particles (Supplementary information, Fig. S1a). The C8 symmetry was applied throughout this stage of data processing. The size and structural features of the NR from *X. laevis* NPC are similar to those from human NPC (Supplementary information, Fig. S1b).

### Data processing and reconstruction of the NR subunit

We extracted the NR subunit particles based on the alignment parameters of the 22-Å NR reconstruction. We updated the orientation, shift and defocus parameters and performed particle re-centering for each subunit according to a published protocol.^23^ 4,411,036 particles of the NR subunit were extracted using a box size of 128 and a binned pixel size of 5.548 Å (Supplementary information, Fig. S2). We performed one round of 3D classification (K = 1) with 10 iterations. The data star file from iteration 10 was then used for re-extraction of bin2 particles with a box size of 256 and a binned pixel size of 2.774 Å (Supplementary information, Fig. S2). The entire dataset of bin2 particles of the IR subunit were then subjected to three rounds of parameter refinement to refine the per-particle angular, shift and local defocus parameters.^24^ This practice allowed selection of 813,020 particles, which yielded a reconstruction of the NR subunit at an average resolution of 5.6 Å. The angular distribution appears to be reasonable (Supplementary information, Fig. S3a). The directional FSC curve and directional histograms for cryo-EM reconstruction of the NR subunit were calculated using a published protocol^46^ (Supplementary information, Fig. S3b). The EM maps display clear features for identification of secondary structural elements (Supplementary information, Figs. S4–S7, S11, S13).

### Atomic modeling of the NR subunit

The atomic coordinates of *X. laevis* CR (PDB: 7FIK)^24^ were manually fitted into our 5.6-Å reconstruction of the *X. laevis* NR subunit using Chimera.^47^ To assist analysis of the EM maps and assignment of the secondary structural elements, we generated sequence alignment^48,49^ for ELYS (Supplementary information, Fig. S8), Nup133 (Supplementary information, Fig. S9) and Nup98 (Supplementary information, Fig. S10). Secondary structural elements of Nup160, Nup37, Nup85, Seh1, Nup43, Nup96, Sec13, Nup107 and Nup133 were assigned on the basis of the structures of these proteins in the CR subunit.^24^ Secondary structural elements of Nup205 and Nup93 were assigned on the basis of the structures of Nup205 and Nup93 in the IR subunit.^25^ The EM density maps allowed unambiguous assignment of most NR components and placement of secondary structural elements. This practice allows identification of 23 molecules of nucleoporins in each NR subunit, including ten molecules each in inner and outer Y complexes (Nup85, Nup160, Nup96, Nup107, Nup133, Nup43, Nup37, Seh1, Sec13, ELYS), one molecule each for Nup205 and Nup93, and one molecule of Nup155 from the IR subunit (Supplementary information, Table S2). Nup155 from the IR subunit is not included in the total count of net protein copies of the NR subunit.

The atomic coordinates of the inner and outer Y complexes, Nup93, and Nup205 from the CR subunit^24^ were docked into the EM maps with the secondary structure elements manually adjusted. The model of Nup155 was generated from the IR subunit^25^ and docked into the EM maps. Using the recently released structure prediction tool AlphaFold,^26^ we generated the atomic coordinates for *X. laevis* ELYS. The predicted structure was docked into the EM maps and individual secondary structure elements were manually adjusted using Coot^50^ based on the 5.6 Å reconstruction of the NR subunit. The final atomic model of the NR subunit contains 18,894 amino acids.

For more information about materials and methods, please see Supplementary information, Data S1.

## Supplementary information

Supplementary information, Figure S1

Supplementary information, Figure S2

Supplementary information, Figure S3

Supplementary information, Figure S4

Supplementary information, Figure S5

Supplementary information, Figure S6

Supplementary information, Figure S7

Supplementary information, Figure S8

Supplementary information, Figure S9

Supplementary information, Figure S10

Supplementary information, Figure S11

Supplementary information, Figure S12

Supplementary information, Figure S13

Supplementary information, Table S1

Supplementary information, Table S2

Supplementary information, Data S1

## Data Availability

The atomic coordinates of the NR subunit have been deposited in the Protein Data Bank with the accession code 7WB4. The EM map for the NR subunit has been deposited in the EMDB with the accession code EMD-32394.
