# Supplementary material for: Cryo-EM structure of the nuclear ring from Xenopus laevis nuclear pore complex
Source: Cell Res. 2022 Feb 17;32(4):349–58. doi: 10.1038/s41422-021-00610-w (PMC8976044; doi:10.1038/s41422-021-00610-w)
Supplement: Supplementary file 1 — Supplementary information, Figure S1 [file 41422_2021_610_MOESM1_ESM.pdf]

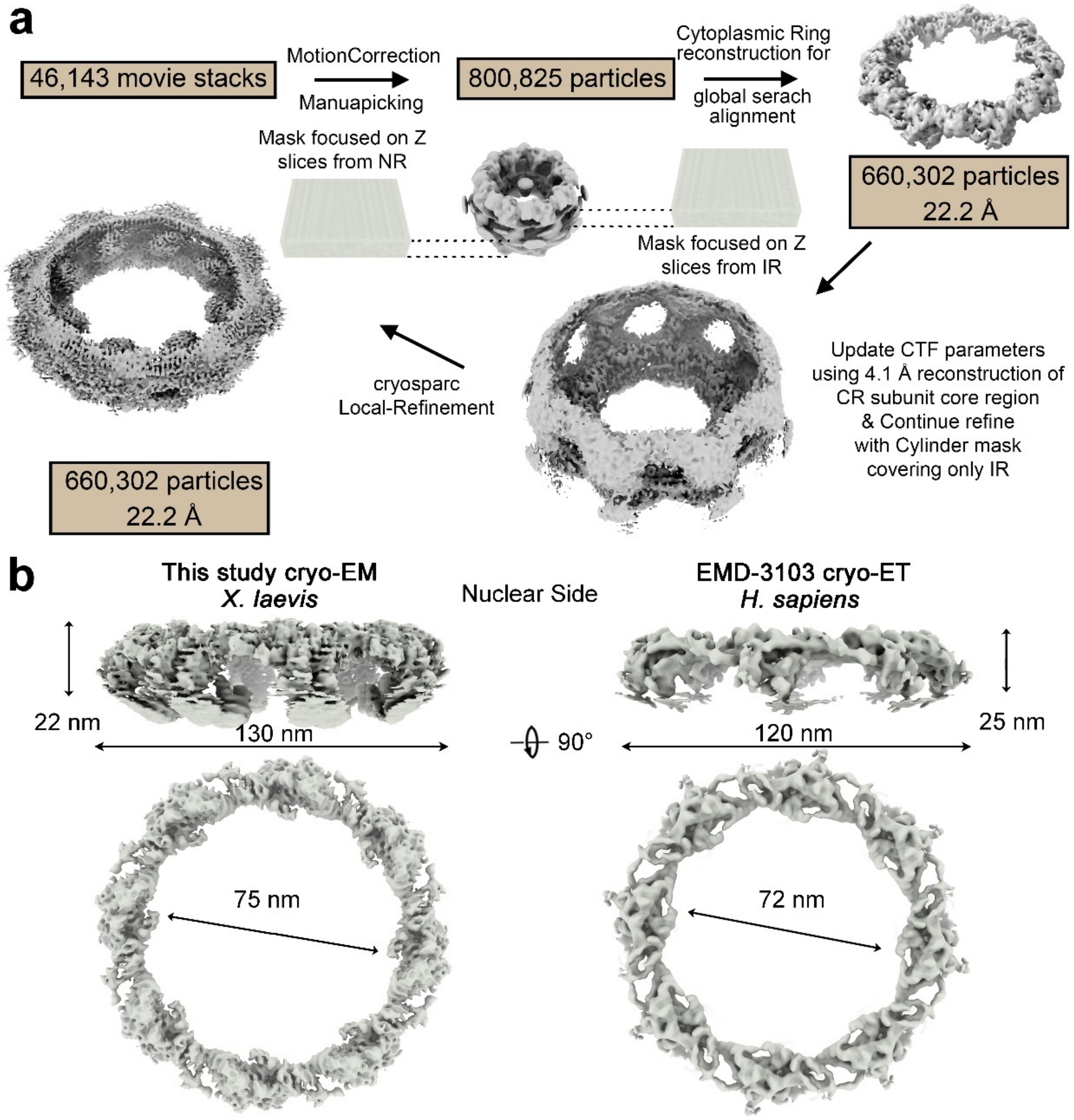

Supplementary information, Fig. S1 | Cryo-EM analysis for the NR of NPC from *X. laevis* oocytes.

a, A flowchart of data analysis for reconstruction of the NR from *X. laevis* NPC at 22 Å resolution. **b**, Comparison of overall structural features between the NR of *X. laevis* NPC and the NR of human NPC[1]. Notably, the experimental methods for structure determination are different.

[1]von Appen, A. *et al*. In situ structural analysis of the human nuclear pore complex. *Nature* **526**, 140-143, doi:10.1038/nature15381 (2015).
