# Supplementary material for: Cryo-EM structure of the nuclear ring from Xenopus laevis nuclear pore complex
Source: Cell Res. 2022 Feb 17;32(4):349–58. doi: 10.1038/s41422-021-00610-w (PMC8976044; doi:10.1038/s41422-021-00610-w)
Supplement: Supplementary file 2 — Supplementary information, Figure S2 [file 41422_2021_610_MOESM2_ESM.pdf]

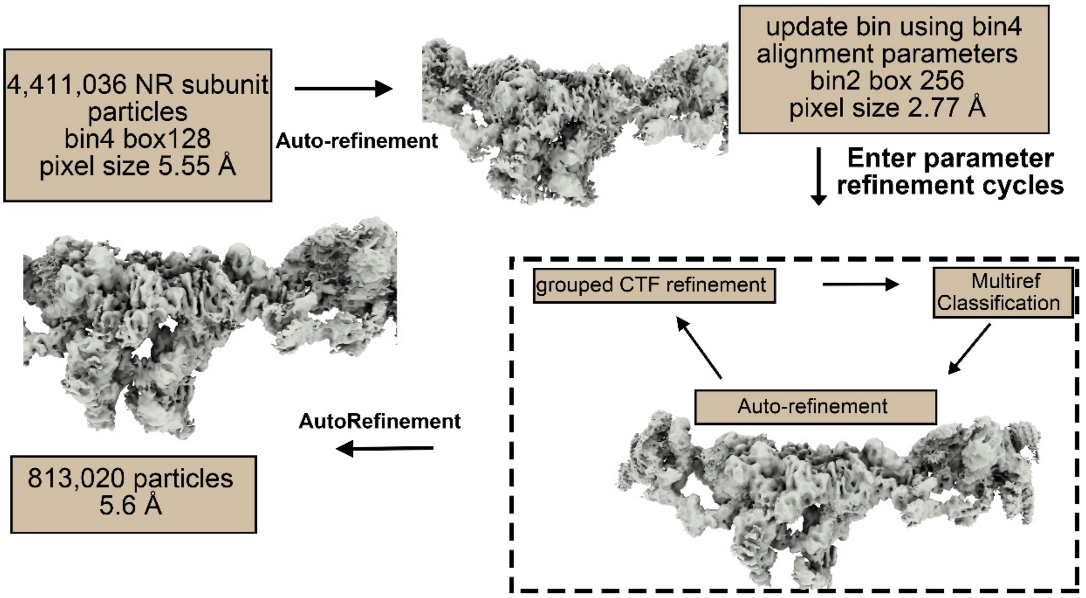

Supplementary information, Fig. S2 | Processing of the cryo-EM data for reconstruction of the NR subunit from *X. laevis* NPC.

Shown here is a flowchart of data processing for reconstruction of the NR subunit to an average resolution of 5.6 Å. This part of the data analysis involves the bin-4 and bin-2 levels.
