# Supplementary material for: Cryo-EM structure of the nuclear ring from Xenopus laevis nuclear pore complex
Source: Cell Res. 2022 Feb 17;32(4):349–58. doi: 10.1038/s41422-021-00610-w (PMC8976044; doi:10.1038/s41422-021-00610-w)
Supplement: Supplementary file 3 — Supplementary information, Figure S3 [file 41422_2021_610_MOESM3_ESM.pdf]

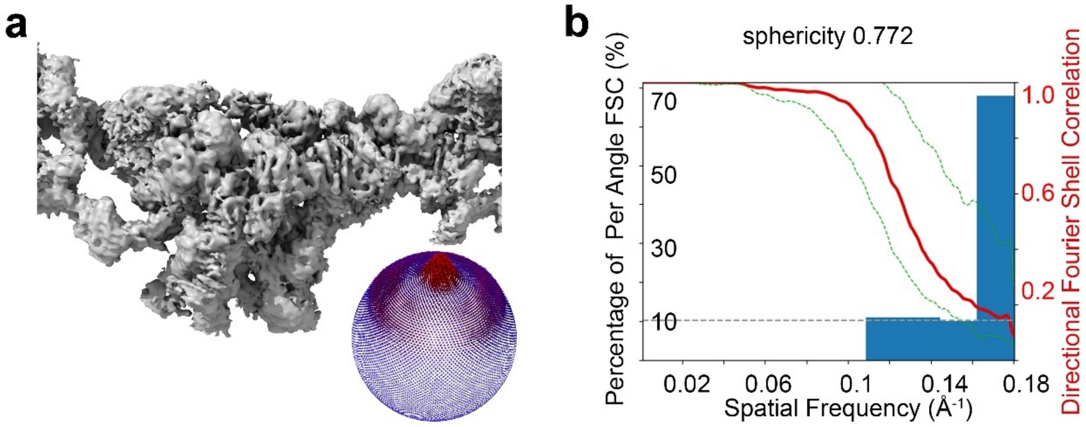

Supplementary information, Fig. S3 | Cryo-EM analysis of the NR subunit.
a, Angular distribution of the single-particle cryo-EM reconstruction for the NR subunit. Each cylinder represents one view and the height of the cylinder is proportional to the number of particles for that view. **b**, Directional Fourier Shell correlation (FSC) curves and directional FSC histograms for cryo-EM reconstruction of the NR subunit. All directional FSC curves were calculated using the following website: https://3dfsc.salk.edu[1].

[1]Tan, Y. Z. *et al*. Addressing preferred specimen orientation in single-particle cryo-EM through tilting. *Nat Methods* **14**, 793-796, doi:10.1038/nmeth.4347 (2017).
