# Supplementary material for: Cryo-EM structure of the nuclear ring from Xenopus laevis nuclear pore complex
Source: Cell Res. 2022 Feb 17;32(4):349–58. doi: 10.1038/s41422-021-00610-w (PMC8976044; doi:10.1038/s41422-021-00610-w)
Supplement: Supplementary file 4 — Supplementary information, Figure S4 [file 41422_2021_610_MOESM4_ESM.pdf]

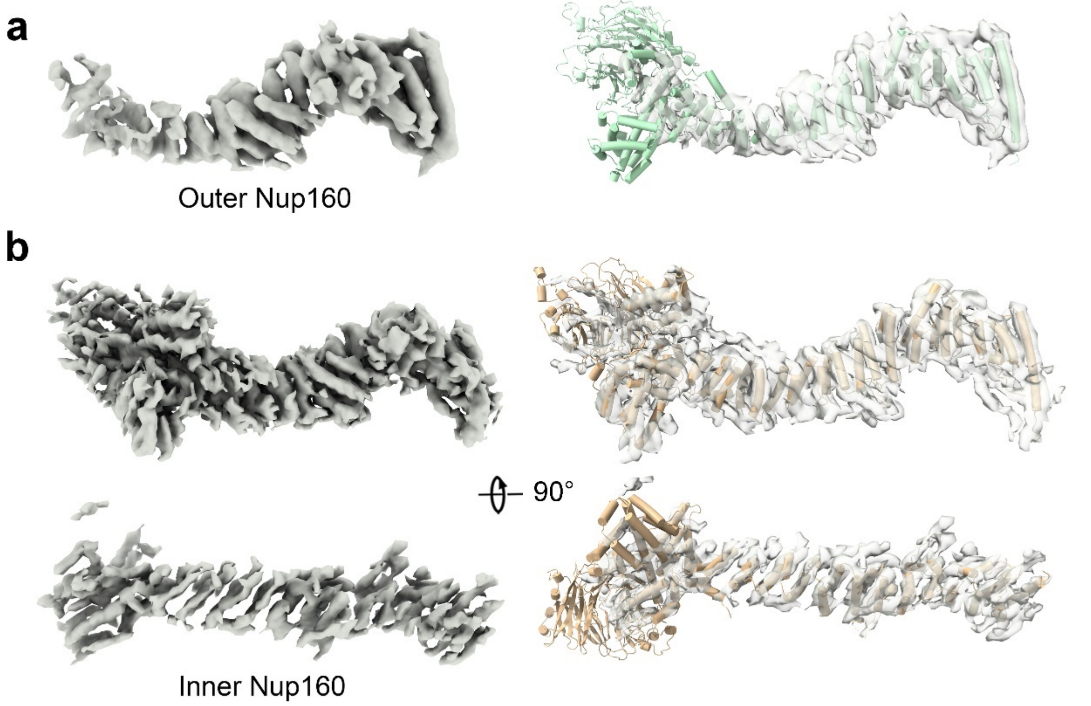

Supplementary information, Fig. S4 | The EM density maps for Nup160.

a, The overall EM density map of outer Nup160. The original EM map with and without structure docking is shown in the right and left panels, respectively. **b**, The overall EM density map of inner Nup160. The original EM map is shown in two left panels, which are related by a 90-degree rotation. The EM map with structure docking is shown in two right panels. All EM density maps in this and following figures were prepared using the reconstruction of the NR subunit with a contour level between 5σ and 10σ.
