# Supplementary material for: Cryo-EM structure of the nuclear ring from Xenopus laevis nuclear pore complex
Source: Cell Res. 2022 Feb 17;32(4):349–58. doi: 10.1038/s41422-021-00610-w (PMC8976044; doi:10.1038/s41422-021-00610-w)
Supplement: Supplementary file 5 — Supplementary information, Figure S5 [file 41422_2021_610_MOESM5_ESM.pdf]

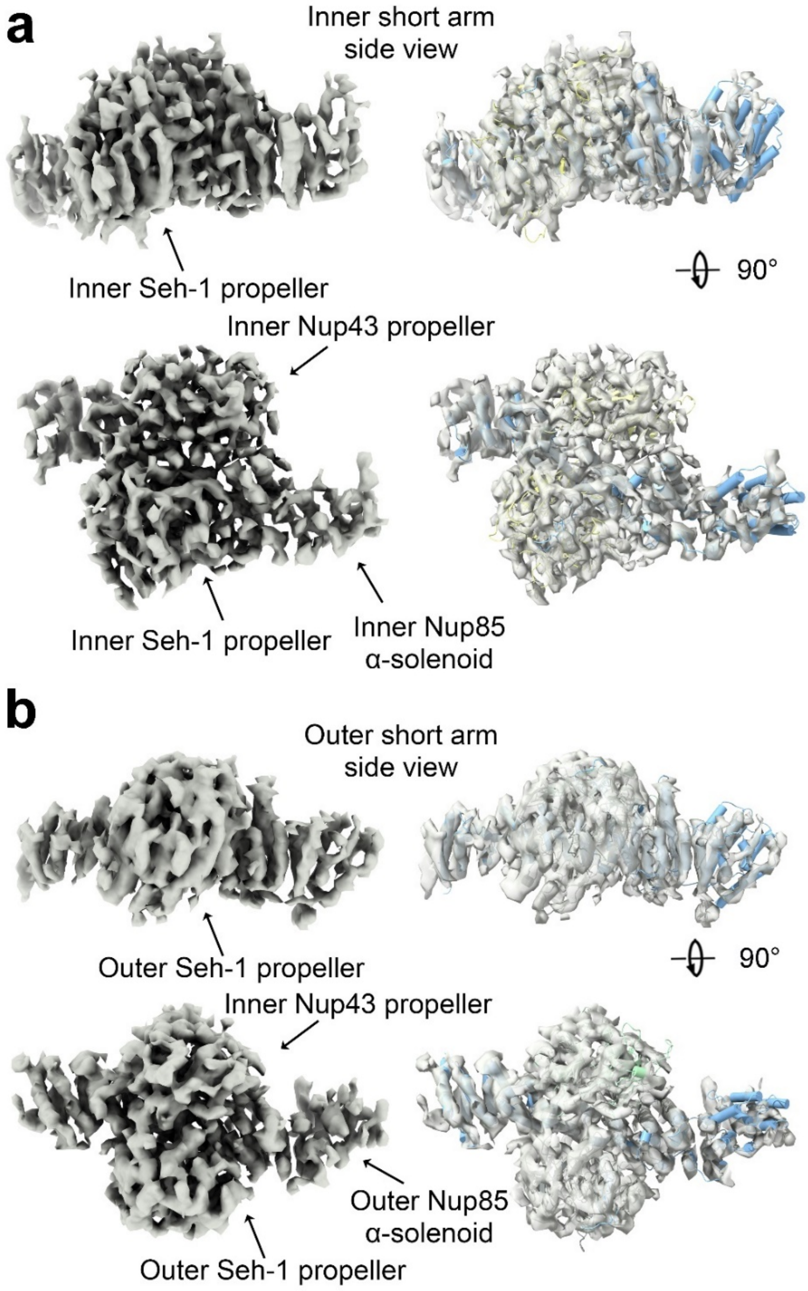

Supplementary information, Fig. S5 | The EM density maps for the short arm regions of the Y complexes.

a, The overall EM density map for the short arm of inner Y complex. The original EM map is shown in two left panels, which are related by a 90-degree rotation. The EM map with structure docking is shown in two right panels. **b**, The overall EM density map for the short arm of outer Y complex. The original EM map is shown in two left panels, which are related by a 90-degree rotation. The EM map with structure docking is shown in two right panels.
