# Supplementary material for: Cryo-EM structure of the nuclear ring from Xenopus laevis nuclear pore complex
Source: Cell Res. 2022 Feb 17;32(4):349–58. doi: 10.1038/s41422-021-00610-w (PMC8976044; doi:10.1038/s41422-021-00610-w)
Supplement: Supplementary file 6 — Supplementary information, Figure S6 [file 41422_2021_610_MOESM6_ESM.pdf]

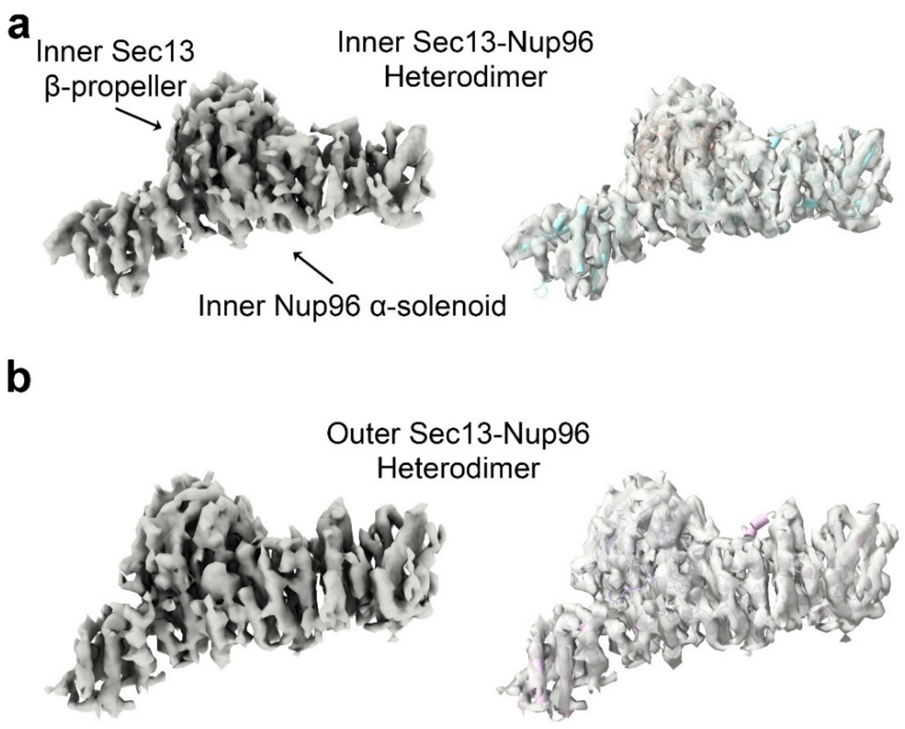


**Supplementary information, Fig. S6 | The EM density maps for the Nup96-Sec13 heterodimer.**

**a**, The overall EM density map for the Nup96-Sec13 heterodimer of the inner Y complex. The original EM map with and without structure docking is shown in the right and left panels, respectively. **b**, The overall EM density map for the Nup96-Sec13 heterodimer of the outer Y complex. The original EM map with and without structure docking is shown in the right and left panels, respectively.
