# Supplementary material for: Cryo-EM structure of the nuclear ring from Xenopus laevis nuclear pore complex
Source: Cell Res. 2022 Feb 17;32(4):349–58. doi: 10.1038/s41422-021-00610-w (PMC8976044; doi:10.1038/s41422-021-00610-w)
Supplement: Supplementary file 7 — Supplementary information, Figure S7 [file 41422_2021_610_MOESM7_ESM.pdf]

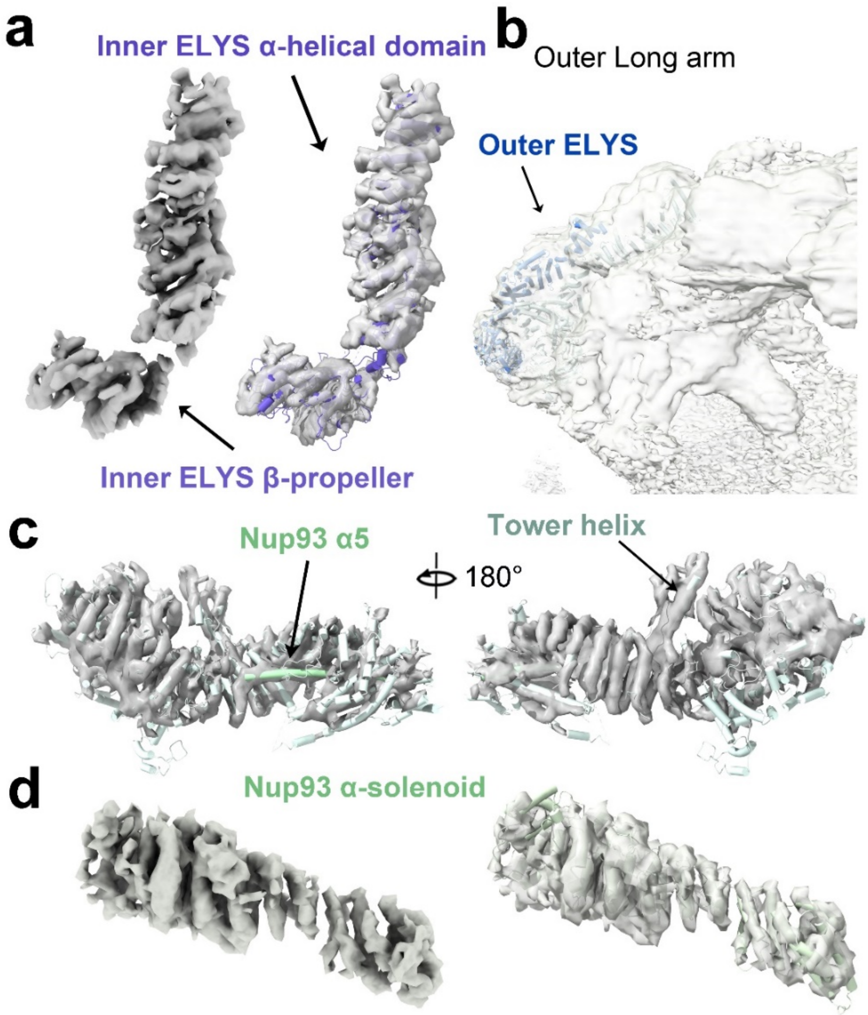

Supplementary information, Fig. S7 | The EM density maps for ELYS, Nup205, and Nup93 of the NR subunit.

a, The overall EM density map of inner ELYS. The original EM map with and without structure docking is shown in the right and left panels, respectively. The structure of *X. laevis* ELYS was generated using AlphaFold[1]. **b**, The overall EM density map of outer ELYS. Due to its peripheral location, this part of the EM map is of lower resolution. Nonetheless, low pass filtered EM map still clearly shows the existence of outer ELYS, which binds to outer Nup160 with an overall binding pose similar to that of inner ELYS. **c**, The overall EM density map of Nup205. The original EM map with structure docking is shown in two related views. The extended helix α5 from Nup93 is included in the structure. **d**, The overall EM density map of the Nup93 α-solenoid. The original EM map with and without structure docking is shown in the right and left panels, respectively.

[1]Jumper, J. *et al*. Highly accurate protein structure prediction with AlphaFold. *Nature* **596**, 583-589, doi:10.1038/s41586-021-03819-2 (2021).
