# Supplementary material for: Cryo-EM structure of the nuclear ring from Xenopus laevis nuclear pore complex
Source: Cell Res. 2022 Feb 17;32(4):349–58. doi: 10.1038/s41422-021-00610-w (PMC8976044; doi:10.1038/s41422-021-00610-w)
Supplement: Supplementary file 10 — Supplementary information, Figure S10 [file 41422_2021_610_MOESM10_ESM.pdf]

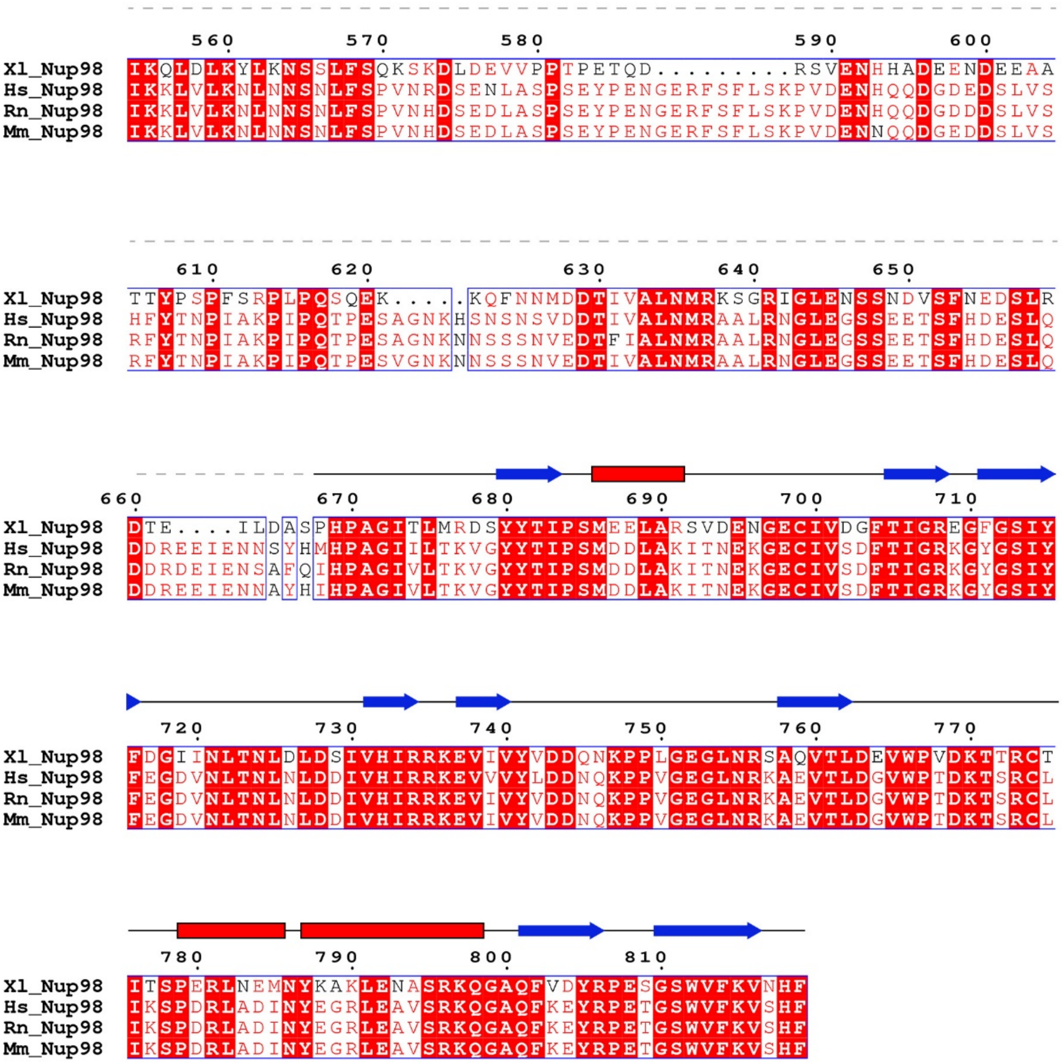

Supplementary information, Fig. S10 | Sequence alignment around the autoproteolytic domain (APD) among Nup98 orthologues from multiple vertebrates.

The sequence alignment of Nup98 from *X. laevis* (Xl), *Homo sapiens* (Hs), *Rattus norvegicus* (Rn), and *Mus musculus* (Mm) is shown around the Nup98 autoproteolytic domain. Conserved amino acids are boxed. Invariant residues are highlighted in red background. The observed secondary structural elements are indicated above the sequences.
