# Supplementary material for: Cryo-EM structure of the nuclear ring from Xenopus laevis nuclear pore complex
Source: Cell Res. 2022 Feb 17;32(4):349–58. doi: 10.1038/s41422-021-00610-w (PMC8976044; doi:10.1038/s41422-021-00610-w)
Supplement: Supplementary file 11 — Supplementary information, Figure S11 [file 41422_2021_610_MOESM11_ESM.pdf]

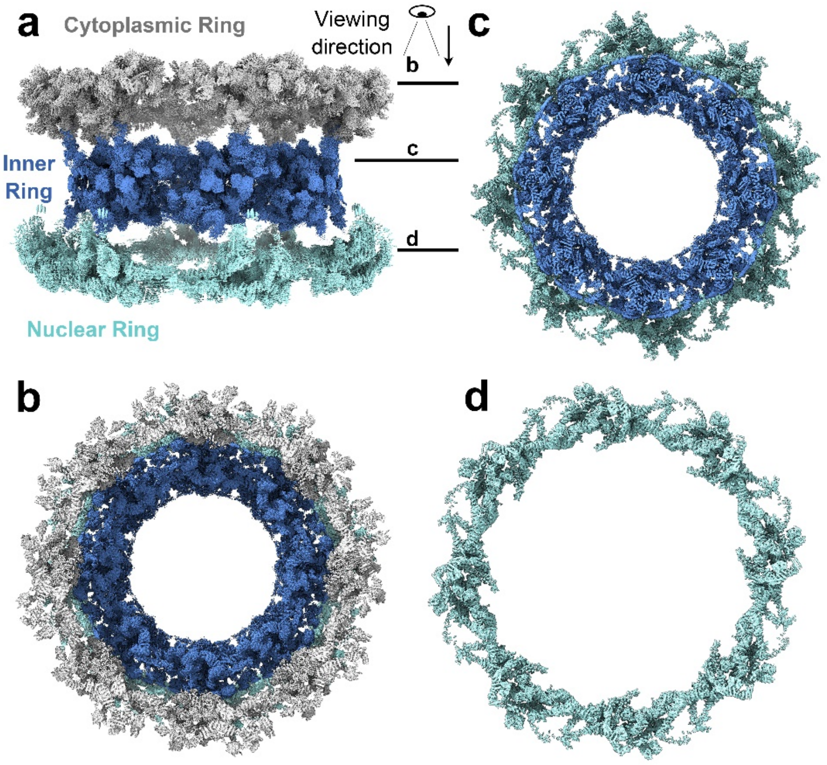

Supplementary information, Fig. S11 | The composite EM density maps for the central ring scaffold of the *X. laevis* NPC.

a, A side view of the composite EM density map for the central ring scaffold of the *X. laevis* NPC. The CR and the NR are above and below the central IR, respectively. **b**, A central slice of the NPC EM map along the nucleocytoplasmic axis. This view is along the nucleocytoplasmic axis from the cytoplasmic side. This slice includes a portion of the CR, the entire IR, and the entire NR. **c**, A slice of the NPC EM map along the nucleocytoplasmic axis. This slice includes a portion of the IR and the entire NR. **d**, A slice of the NPC EM map along the nucleocytoplasmic axis. This slice includes a portion of the NR.
