# Supplementary material for: Cryo-EM structure of the nuclear ring from Xenopus laevis nuclear pore complex
Source: Cell Res. 2022 Feb 17;32(4):349–58. doi: 10.1038/s41422-021-00610-w (PMC8976044; doi:10.1038/s41422-021-00610-w)
Supplement: Supplementary file 12 — Supplementary information, Figure S12 [file 41422_2021_610_MOESM12_ESM.pdf]

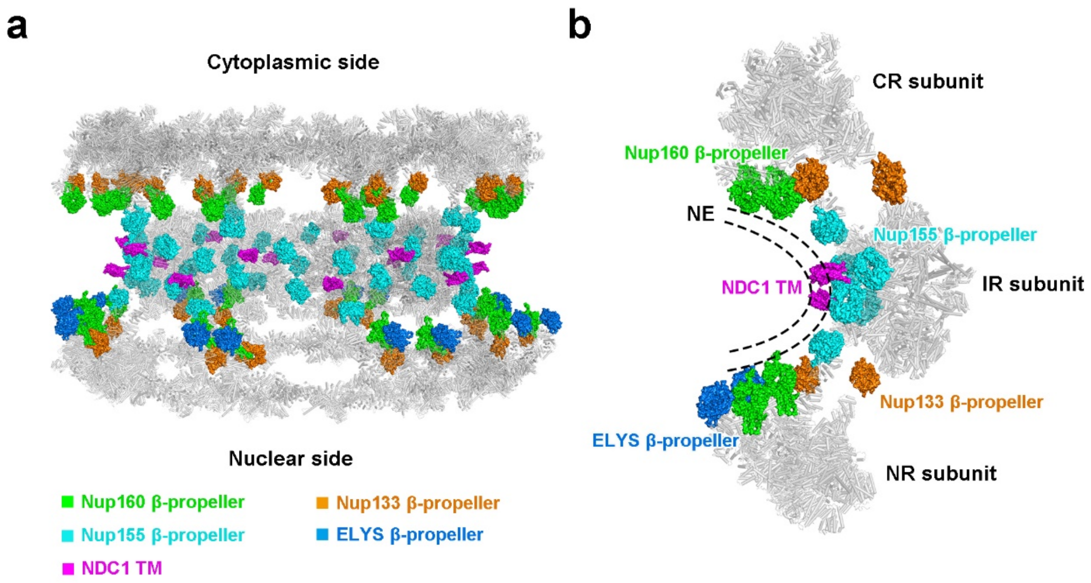

Supplementary information, Fig. S12 | Structure motifs of the *X. laevis* NPC that contact the NE.

a, An overall view of the *X. laevis* NPC. The structure motifs that contact the NE are shown in color-coded surface representation. These motifs include the β-propeller domains from Nup160 (green), Nup155 (cyan), Nup133 (orange) and ELYS (marine) and the transmembrane domain (TM) of NDC1 (magenta). **b**, An overall view of one subunit of the *X. laevis* NPC. This subunit contains one subunit each of the CR, IR, and NR. The structure motifs that contact the NE are shown in color-coded surface representation. The subunit is anchored on the NE mainly through the TM domain of NDC1 and the β-propeller domains from Nup155, Nup160, Nup133, and ELYS.
