# Supplementary material for: Cryo-EM structure of the nuclear ring from Xenopus laevis nuclear pore complex
Source: Cell Res. 2022 Feb 17;32(4):349–58. doi: 10.1038/s41422-021-00610-w (PMC8976044; doi:10.1038/s41422-021-00610-w)
Supplement: Supplementary file 13 — Supplementary information, Figure S13 [file 41422_2021_610_MOESM13_ESM.pdf]

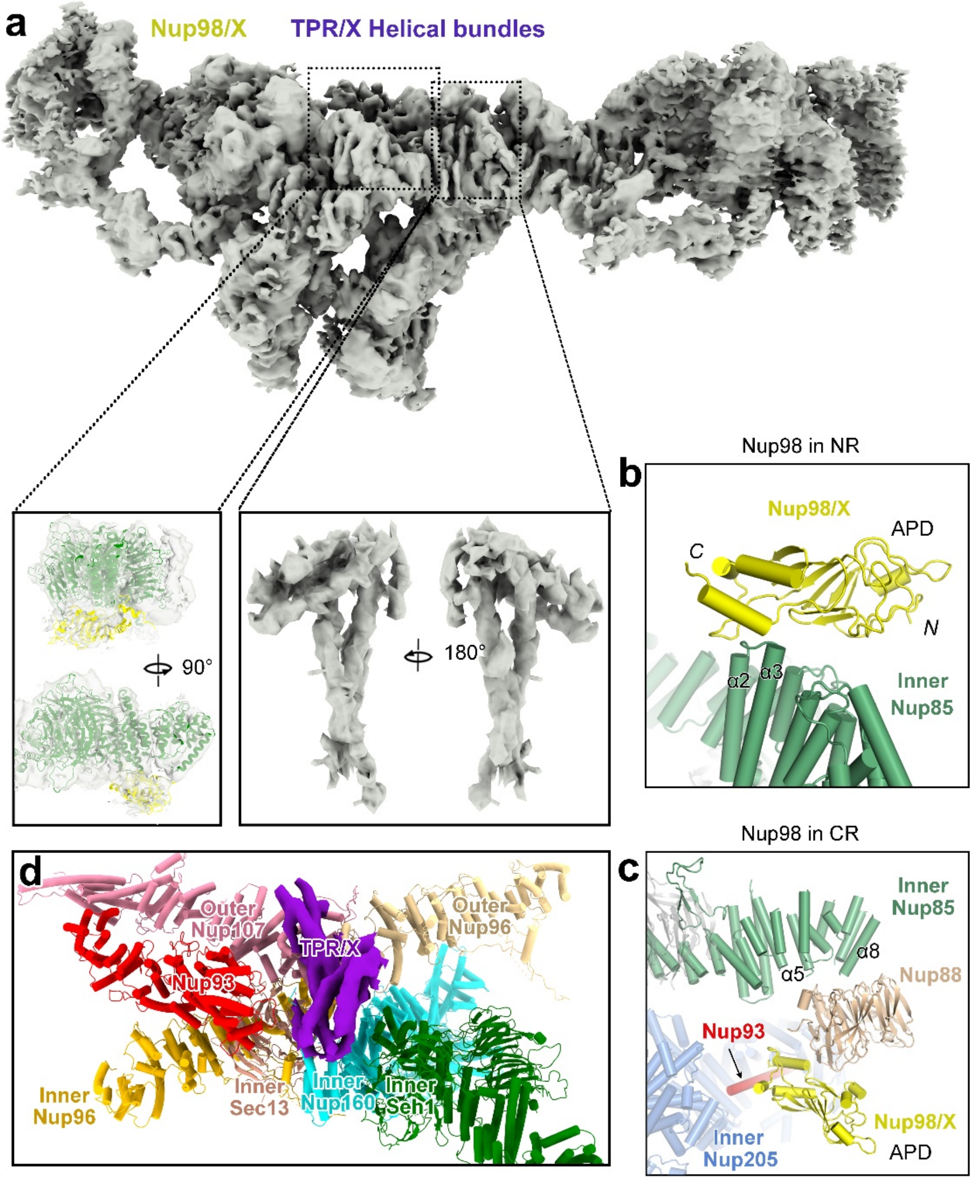

Supplementary information, Fig. S13 | The EM density maps for candidate Nup98 and TPR proteins of the NR subunit.

a, The overall EM density map of the NR subunit. The putative locations for Nup98 and TPR are boxed. Close-up views of the putative locations for Nup98 and TPR on the local EM density are shown. **b**, A close-up view on the interactions between Nup98/X and inner Nup85. The C-terminal region of Nup98 autoproteolytic domain (APD) may contact two helices α2/α3 of inner Nup85. **c**, A close-up view on Nup98/X and its surrounding proteins in the CR subunit[1]. The APD of Nup98 in CR interacts with the β-propeller of Nup88, which then binds inner Nup85. Specifically, the β-propeller of Nup88 contacts the ridges of two helices α5/α8 of inner Nup85. In addition, the APD of Nup98 may contact the extend helix α5 of Nup93. **d**, The helical bundles of putative TPR/X interact with the long arm of the inner Y complex and the stem of outer Y complex.

[1]Zhu, X. *et al*. Near-atomic Structure of the Cytoplasmic Ring of the *Xenopus laevis* Nuclear Pore Complex. In press (2021).
