# Supplementary material for: Cryo-EM structure of the nuclear ring from Xenopus laevis nuclear pore complex
Source: Cell Res. 2022 Feb 17;32(4):349–58. doi: 10.1038/s41422-021-00610-w (PMC8976044; doi:10.1038/s41422-021-00610-w)
Supplement: Supplementary file 15 — Supplementary information, Table S2 [file 41422_2021_610_MOESM15_ESM.pdf]

**Supplementary information, Table S2 | Summary of model building for the NR subunit of *X. laevis* NPC.**

| | Molecule **Vertebrates/** Yeast | Copy No. | Length *Xenopus* | UniProt No. *Xenopus* | PDB code | Modeling | Model length | Resolution (Å) | Chain ID |
|---|---|---|---|---|---|---|---|---|---|
| **Y complex (S1)** | **Nup85**/Nup85 | 2 | 653 | Q68FJ0 | 7FIK/AF | RD | ~630 | 6.5~8.0 | B/b |
| | **Nup160**/Nup120 | 2 | 1435 | A0A1L8GIX3 | 7FIK/AF | RD | ~1400 | 6.5~8.0 | E/e |
| | **Nup96**/Nup145C | 2 | 923 | A0A1L8HBE3 | 7FIK/AF | RD | ~700 | 6.5~7.5 | G/g |
| | **Nup107**/Nup84 | 2 | 916 | A2RV69 | 7FIK/AF | RD | ~800 | 6.5~8.5 | I/i |
| | **Nup133**/Nup133 | 2 | 1140 | A0A1L8H1I9 | 7FIK/AF | RD | ~1000 | 7.5~9.0 | J/j |
| | **Sec13**/Sec13 | 2 | 320 | Q7ZYJ8 | 7FIK/AF | RD | ~300 | 6.5~7.5 | H/h |
| | **Seh1**/Seh1 | 2 | 360 | Q4FZW5 | 7FIK/AF | RD | ~310 | 7.0~8.5 | D/d |
| | **Nup43**/- | 2 | 375 | Q05AW3 | 7FIK/AF | RD | ~340 | 7.0~8.5 | C/c |
| | **Nup37**/Nup37 | 2 | 326 | Q66IZ6 | 7FIK/AF | RD | ~320 | 7.0~8.5 | F/f |
| **ELYS (S1)** | **ELYS/-** | 2 | 2408 | Q5U249 | AF | RD | ~1000 | 7.5~9.0 | N/n |
| **Nup205 (S1)** | **Nup205**/Nup192 | 1 | 2011 | Q642R6 | 7FIK | RD | ~2000 | 7.0~8.5 | A |
| **Nup93 (S1)** | **Nup93**/Nic96 | 1 | 820 | Q7ZX96 | 7FIK | RD | ~700 | 7.0~8.5 | L |
| **Nup155 (S1)** | **Nup155**/Nup155 | 1 | 1388 | Q7ZWL0 | AF | RD | ~330 | 8.0~10.0 | K |
| **Nup93 (S2)** | **Nup93**/Nic96 | 1 | 820 | Q7ZX96 | AF | RD | ~80 | 6.5~8.0 | O |
| **Nup107 (S2)** | **Nup107**/Nup84 | 2 | 916 | A2RV69 | 7FIK/AF | RD | ~800 | 6.5~8.5 | M |
| **Nup133 (S2)** | **Nup133**/Nup133 | 2 | 1140 | A0A1L8H1I9 | 7FIK/AF | RD | ~1000 | 7.0~8.5 | P/p |

Under the column labeled "Molecule", proteins from vertebrates and yeasts are shown, respectively, with vertebrate components in bold. Under the column labeled "PDB code", AF stands for AlphaFold (model generated from AlphaFold prediction). Under the column labeled "Modeling", RD stands for rigid docking and manual adjustment.
