# Supplementary material for: Cryo-EM structure of the nuclear ring from Xenopus laevis nuclear pore complex
Source: Cell Res. 2022 Feb 17;32(4):349–58. doi: 10.1038/s41422-021-00610-w (PMC8976044; doi:10.1038/s41422-021-00610-w)
Supplement: Supplementary file 16 — Supplementary information, Data S1 [file 41422_2021_610_MOESM16_ESM.pdf]

# Supplementary information, Data S1

## Materials and methods

### Cryo-EM sample preparation and EM data acquisition

The same cryo-EM dataset used for reconstruction of the cytoplasmic ring (CR)[1] and inner ring (IR)[2] from *Xenopus laevis* (*X. laevis*) oocytes was used for reconstruction of the nuclear ring (NR) in this study. In short, the nuclear envelope (NE) from *X. laevis* oocytes was prepared as previously described[3,4]. The gold EM grids (R1.2/1.3, R2/1, and R2/2; Quantifoil, Jena, Germany) were blotted for 8 seconds with a blot force of 15 and vitrified by plunge-freezing into liquid ethane using Vitrobot Mark IV (Thermo Fisher Scientific) at 8 °C under 100% humidity.

Details of data acquisition are as described, with the grids tilting at angles of 0, 30, 45, and 55 degrees[1]. A dataset of 46,143 micrographs were recorded on a Titan Krios electron microscope (FEI) operating at 300 kV with a nominal magnification of 64,000x and equipped with a Gatan GIF Quantum energy filter (slit width 20 eV) (Supplementary information, Table S1). A K3 detector (Gatan Company) operating at the super-resolution mode was used for data acquisition, with a calibrated pixel size of 0.6935 Å for the movie files (Supplementary information, Table S1). The movie images were binned twice during motion correction, resulting in a final pixel size of 1.387 Å for the motion corrected images. All frames in each stack were aligned and summed using MotionCor2[5]. During the alignment process, the raw frames were divided into 13x11 patches to perform local alignment and polynomial model estimation[6]. Dose weighting was performed using MotionCor2[5]. The average defocus

values were set between -1.5 and -3.0 μm and per micrograph CTF parameters were estimated using Gctf[7].

**An initial model of the NR from *X. laevis* NPC**

33,747 micrographs were manually selected from the original dataset of 46,143 micrographs for further processing. A total of 800,825 particles were manually selected from these micrographs (Supplementary information, Fig. S1a). Initial per-particle local defocus estimation was carried out as previously described[4] prior to all other data processing procedures.

The central portion of an NPC comprises four ring scaffolds: CR, IR, NR, and luminal ring (LR). Due to the inherent flexibility among the four rings, it is practically impossible to refine the entire NPC as a single particle to high resolution. We therefore carried out initial pose estimation of the NPC particles on one of the relatively stable ring scaffolds: the CR. The NPC particles were first aligned to the CR side as described[1]. This procedure allowed selection of 660,302 NPC particles that contributed to the final reconstruction. Following refinement of the CR structure with these 660,302 NPC particles, we further refined the EM density that belongs to other rings using a confined angular and shift search range. We first continued the 3D refinement procedure from the last iteration with a layered mask focusing on the IR and LR layer (the layer immediately adjacent to the CR layer). Only pixels within a specific layer, with $z\_start < z < z\_end$, have pixel values of 1; all other pixels that have z coordinates below or above this layer have zero values. Transition between

these two regions follows a raised cosine scheme. The continue refinement yielded a reconstruction of the IR at 22 Å resolution based on 660,302 NPC particles. The same local refinement strategy was applied again to help refine the EM density that belongs to the NR. Continuing from 3D auto-refinement of the IR, a layered mask focusing on the NR layer was further applied for 3D refinement. A final reconstruction of the NR was finally obtained out of 660,302 NPC particles (Supplementary information, Fig. S1a). The C8 symmetry was applied throughout this stage of data processing. The size and structural features of the NR from *X. laevis* NPC are similar to those from human NPC (Supplementary information, Fig. S1b).

**Data processing and reconstruction of the NR subunit**

We extracted the NR subunit particles based on the alignment parameters of the 22-Å NR reconstruction. We updated the orientation, shift and defocus parameters and performed particle re-centering for each subunit according to a published protocol[4]. 4,411,036 particles of the NR subunit were extracted using a box size of 128 and a binned pixel size of 5.548 Å (Supplementary information, Fig. S2). We performed one round of 3D classification (K=1) with 10 iterations. The data star file from iteration 10 was then used for re-extraction of bin2 particles with a box size of 256 and a binned pixel size of 2.774 Å (Supplementary information, Fig. S2). The entire dataset of bin2 particles of the IR subunit were then subjected to three rounds of parameter refinement to refine the per-particle angular, shift and local defocus parameters[1]. This practice allowed selection of 813,020 particles, which yielded a

reconstruction of the NR subunit at an average resolution of 5.6 Å. The angular distribution appears to be reasonable (Supplementary information, Fig. S3a). The directional Fourier shell correlation (FSC) curve and directional histograms for cryo-EM reconstruction of the NR subunit were calculated using a published protocol[8] (Supplementary information, Fig. S3b). The EM maps display clear features for identification of secondary structural elements (Supplementary information, Figs. S4-S7, Fig. S11, Fig. S13).

**Atomic modeling of the NR subunit**

The atomic coordinates of *X. laevis* CR (PDB: 7FIK)[1] were manually fitted into our 5.6-Å reconstruction of the *X. laevis* NR subunit using Chimera[9]. To assist analysis of the EM maps and assignment of the secondary structural elements, we generated sequence alignment[10,11] for ELYS (Supplementary information, Fig. S8), Nup133 (Supplementary information, Fig. S9) and Nup98 (Supplementary information, Fig. S10). Secondary structural elements of Nup160, Nup37, Nup85, Seh1, Nup43, Nup96, Sec13, Nup107 and Nup133 were assigned on the basis of the structures of these proteins in the CR subunit[1]. Secondary structural elements of Nup205 and Nup93 were assigned on the basis of the structures of Nup205 and Nup93 in the IR subunit[2]. The EM density maps allowed unambiguous assignment of most NR components and placement of secondary structural elements. This practice allows identification of 23 molecules of nucleoporins in each NR subunit, including ten molecules each in inner and outer Y complexes (Nup85, Nup160, Nup96, Nup107,

Nup133, Nup43, Nup37, Seh1, Sec13, ELYS), one molecule each for Nup205 and Nup93, and one molecule of Nup155 from the IR subunit (Supplementary information, Table S2). Nup155 from the IR subunit is not included in the total count of net protein copies of the NR subunit.

The atomic coordinates of the inner and outer Y complexes, Nup93, and Nup205 from the CR subunit[1] were docked into the EM maps with the secondary structure elements manually adjusted. The model of Nup155 was generated from the IR subunit[2] and docked into the EM maps. Using the recently released structure prediction tool AlphaFold[12], we generated the atomic coordinates for *X. laevis* ELYS. The predicted structure was docked into the EM maps and individual secondary structure elements were manually adjusted using Coot[13] based on the 5.6 Å reconstruction of the NR subunit. The final atomic model of the NR subunit contains 18,894 amino acids.

**References:**


1 Zhu, X. *et al*. Near-atomic Structure of the Cytoplasmic Ring of the *Xenopus laevis* Nuclear Pore Complex. In press (2021).

2 Huang, G. *et al*. Cryo-EM Structure of the Inner Ring from *Xenopus laevis* Nuclear Pore Complex. *bioRxiv*, 2021.2011.2013.468242, doi:10.1101/2021.11.13.468242 (2021).

3 Zhang, Y. *et al*. Molecular architecture of the luminal ring of the Xenopus laevis nuclear pore complex. *Cell Res* **30**, 532-540, doi:10.1038/s41422-020-0320-y (2020).

4 Huang, G. *et al*. Structure of the cytoplasmic ring of the Xenopus laevis nuclear pore complex by cryo-electron microscopy single particle analysis. *Cell Res* **30**, 520-531, doi:10.1038/s41422-020-0319-4 (2020).

5 Zheng, S. Q. *et al*. MotionCor2: anisotropic correction of beam-induced motion for improved cryo-electron microscopy. *Nat Methods* **14**, 331-332, doi:10.1038/nmeth.4193 (2017).

6 Zivanov, J. *et al*. New tools for automated high-resolution cryo-EM structure determination in RELION-3. *Elife* **7**, doi:10.7554/eLife.42166 (2018).

7 Zhang, K. Gctf: Real-time CTF determination and correction. *J Struct Biol* **193**, 1-12, doi:10.1016/j.jsb.2015.11.003 (2016).

8 Tan, Y. Z. *et al*. Addressing preferred specimen orientation in single-particle cryo-EM through tilting. *Nat Methods* **14**, 793-796, doi:10.1038/nmeth.4347 (2017).

9 Pettersen, E. F. *et al*. UCSF Chimera--a visualization system for exploratory research and analysis. *J Comput Chem* **25**, 1605-1612, doi:10.1002/jcc.20084 (2004).

10 Madeira, F. *et al*. The EMBL-EBI search and sequence analysis tools APIs in 2019. *Nucleic Acids Res* **47**, W636-W641, doi:10.1093/nar/gkz268 (2019).

11 Robert, X. & Gouet, P. Deciphering key features in protein structures with the new ENDscript server. *Nucleic Acids Res* **42**, W320-324, doi:10.1093/nar/gku316 (2014).

12 Jumper, J. *et al*. Highly accurate protein structure prediction with AlphaFold. *Nature* **596**, 583-589, doi:10.1038/s41586-021-03819-2 (2021).

13 Emsley, P. & Cowtan, K. Coot: model-building tools for molecular graphics. *Acta Crystallogr D Biol Crystallogr* **60**, 2126-2132, doi:10.1107/S0907444904019158 (2004).
